# How multisensory neurons solve causal inference

**DOI:** 10.1073/pnas.2106235118

**Published:** 2021-08-04

**Authors:** Reuben Rideaux, Katherine R. Storrs, Guido Maiello, Andrew E. Welchman

**Affiliations:** ^a^Queensland Brain Institute, University of Queensland, 4072 Brisbane, Australia;; ^b^Department of Psychology, University of Cambridge, CB23EB Cambridge, United Kingdom;; ^c^Department of Experimental Psychology, Justus Liebig University Giessen, 35390 Giessen, Germany

**Keywords:** causal inference, multisensory integration, MSTd, visual and vestibular, deep neural network

## Abstract

Sitting in a static railway carriage can produce illusory self-motion if the train on an adjoining track moves off. While our visual system registers motion, vestibular signals indicate that we are stationary. The brain is faced with a difficult challenge: is there a single cause of sensations (I am moving) or two causes (I am static, another train is moving)? If a single cause, integrating signals produces a more precise estimate of self-motion, but if not, one cue should be ignored. In many cases, this process of causal inference works without error, but how does the brain achieve it? Electrophysiological recordings show that the macaque medial superior temporal area contains many neurons that encode combinations of vestibular and visual motion cues. Some respond best to vestibular and visual motion in the same direction (“congruent” neurons), while others prefer opposing directions (“opposite” neurons). Congruent neurons could underlie cue integration, but the function of opposite neurons remains a puzzle. Here, we seek to explain this computational arrangement by training a neural network model to solve causal inference for motion estimation. Like biological systems, the model develops congruent and opposite units and recapitulates known behavioral and neurophysiological observations. We show that all units (both congruent and opposite) contribute to motion estimation. Importantly, however, it is the balance between their activity that distinguishes whether visual and vestibular cues should be integrated or separated. This explains the computational purpose of puzzling neural representations and shows how a relatively simple feedforward network can solve causal inference.

## Significance

We perceive our environment through multiple independent sources of sensory input. The brain is tasked with deciding whether multiple signals are produced by the same or different events (i.e., solve the problem of causal inference). Here, we train a neural network to solve causal inference by either combining or separating visual and vestibular inputs in order to estimate self- and scene motion. We find that the network recapitulates key neurophysiological (i.e., congruent and opposite neurons) and behavioral (e.g., reliability-based cue weighting) properties of biological systems. We show how congruent and opposite neurons support motion estimation and how the balance in activity between these subpopulations determines whether to combine or separate multisensory signals.

Most biological organisms receive multiple independent sources of sensory input that support perception of the environment (e.g., visual, vestibular, auditory, somatosensory, etc.). A fundamental challenge for the brain is to decide whether multiple signals are produced by the same or different events (i.e., solve the problem of causal inference). Sensory signals are noisy and often ambiguous, so combining multiple signals caused by the same event improves the precision with which the world is perceived. However, erroneously combining signals caused by different events reduces the correspondence between perception and the environment.

**Fig. 1.**
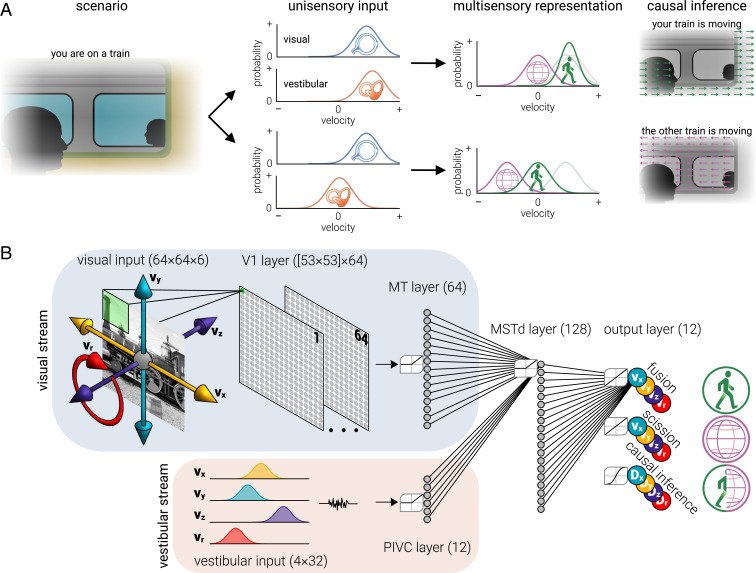
Solving causal inference with a multisensory neural network. (*A*) Two cases (*Top* and *Bottom*) illustrate how causal inference must be solved to estimate movement through the environment. In the first case (*Top*), you are sitting on a train and receive visual and vestibular signals that you are moving forward. These unisensory signals can be represented as probability distributions of velocity. The signals should be combined to produce a more accurate representation of your movement (self-motion, green distribution), it is assumed that the environment is not moving (scene motion, magenta distribution), and you infer the signals were caused by the same event (i.e., your train is moving forward). Note, semitransparent visual and vestibular signals are shown in the “multisensory representation” plot for reference. In the second case (*Bottom*), you receive a visual signal that you are moving forward and a vestibular signal that you are stationary. The signals should be kept separate, self- and scene motion are estimated from vestibular and the difference between vestibular and visual signals, respectively, and you infer that they were caused by different events, (i.e., the other train is moving). (*B*) The network comprised two input streams: visual (blue) and vestibular (orange). The visual stream consisted of an input layer, a convolutional (V1) layer and a fully connected (MT) layer. The vestibular stream consisted of an input layer and a fully connected (PIVC) layer. These streams converged on a common fully connected (MSTd) layer. The activity of the MSTd layer was decoded by four “fusion” regression units to produce estimates of (integrated) self-motion, four “scission” regression units to produce estimates of scene motion, and four binary “causal inference” units to decide whether the motion was produced by the same, or different, events, for each (*v*_*x*_, *v*_*y*_, *v*_*z*_, and *v*_*r*_) axis of velocity. The numbers associated with each layer indicate their dimensions (e.g., there are four vestibular inputs that are each represented by 32 units).

When navigating the environment, many vertebrates receive signals transduced from movement of fluid within the vestibular system, that is, from otolith organs and semicircular canals that detect translational and rotational acceleration of the head, respectively (1, 2). The same navigation also produces uniform changes in the patterns of light that are absorbed by the retina (i.e., optic flow) (3). Both independent sensory signals can be used to infer movement caused by navigation through the environment (i.e., self-motion; Fig. 1, case 1) (4, 5). When the environment is stable, these signals will elicit similar unisensory representations that can be combined to produce more precise representations of self-motion. This improvement in precision can have significant implications; for example, it may be the difference between escaping or being caught by a predator. However, typically the environment is also in motion (e.g., water moving across the ground or clouds across the sky), meaning that visual inputs provide an uncertain combination of self-generated and scene-generated motion (i.e., scene motion; Fig. 1, case 2). In this case, vestibular and visual signals will differ, and self-motion must be inferred only from vestibular signals, while scene motion can be derived from the difference between the two signals. The central nervous system is tasked with deciding when signals are produced by the same or different events (i.e., when to integrate or separate sensory input). Biological systems are capable of automatically performing causal inference with considerable accuracy, yet the neural computations that support this task remain unknown.

Neurophysiological work in macaque monkeys indicates that vestibular and visual signals are combined on the dorsal subdivision of the medial superior temporal area (MSTd) (6–11) and in the ventral intraparietal area (VIP) (12, 13). However, the majority of work has focused on MSTd; refer to ref. 14 for a review. Whether two signals are considered to be caused by the same event determines whether they are combined, thus implicating MSTd as a neural locus for solving this problem. Studies of vestibular–visual integration have identified two types of multisensory neurons with similar prevalence in MSTd and VIP; those that are tuned to vestibular and visual motion in the same direction (congruent neurons) and those that prefer opposite directions (opposite neurons) (6, 8, 11). Congruent neurons appear to combine vestibular–visual signals in a way that assumes a common cause, and their activity is highly predictive of behavioral choice on vestibular–visual combination tasks (8–10). By contrast, the role of opposite neurons remains unclear, as their activity is not predictive of behavioral outcomes on sensory combination tasks. It is possible that opposite neurons support causal inference; however, it is difficult to understand the role of these cells and how they contribute to solving this problem because of limitations imposed by the techniques used to study biological systems.

Here, using an artificial systems approach, we train a neural network to solve causal inference by either combining or separating visual and vestibular inputs in order to estimate self- and scene motion. Our complete access to the artificial system allows unrestricted interrogation of how the problem of causal inference is solved. We previously applied this method to understand unexplained features and reveal characteristics of biological motion processing in areas V1 and MT (15, 16). Here, we apply the same principles to provide an explanation for how visual and vestibular sensory cues that signal motion are processed in MSTd to yield estimates of self- and scene motion and how causal inference is solved by biological systems.

We find that units emerge within the artificial system with properties that match those found in biological systems (i.e., congruent and opposite neurons). Further, the network exhibits key “behavioural” characteristics observed in human and primate studies, such as reliability-based cue weighting (9, 17) and self-motion estimation biased by causal inference (18). We show that while congruent and opposite units primarily support estimation of self- and scene-motion estimation, respectively, activity from both unit types contributes to these representations. Moreover, we find that the balance between congruent and opposite unit activity is used to decide whether to combine or separate visual and vestibular signals (i.e., solve causal inference). These results provide an explanation of how causal inference is solved in biological systems and explains the role of opposite neurons.

## Results

### Network Architecture and Training Protocol.

We created a multisensory artificial system, “MultiNet,” that received visual and vestibular input and was tasked with judging the sources of motion (self- versus scene) to determine whether the inputs should be integrated or separated (Fig. 1*B*). The visual input comprised natural image sequences moving at a range of speeds (±4 pixels/frame) in translational (*x* and *y*), radial (*z*), and rotational (*r*) directions (i.e., rotation around the *z* axis; *SI Appendix*, Fig. S1 *A*, *Top*). The input was convolved with three-dimensional kernels (*x*-*y*-*t*) (“V1 layer”), and the resultant activity was passed to a fully connected layer of units (“MT layer”). The vestibular input comprised four noisy Gaussian distributions to represent signals within vestibular nuclei produced by movement of fluid detected by otolith organs and semicircular canals (19). This input was read-out by a fully connected layer of units (parieto-insular vestibular cortex, “PIVC layer”) (20). The visual and vestibular streams converged on a fully connected layer of units (“MSTd layer”), the activity of which was decoded by an output layer trained to produce estimates of self- and scene motion and perform causal inference. Separate estimates and decisions were produced for horizontal (*v*_*x*_), vertical (*v*_*y*_), motion-in-depth (*v*_*z*_), and rotational velocity (*v*_*r*_). Note that motion-in-depth and rotation produce image speeds that increase with distance from the focal point; thus, here, *v*_*z*_ and *v*_*r*_ refer to the midpoint axial and rotational speed across the image.

We trained MultiNet for 50 epochs on 64,000 randomly generated visual–vestibular input combinations. Within each axis, absolute velocity differences smaller than the median across all combinations were classed as being caused by the same event. Multisensory “fusion” and “scission” units were trained, respectively, to report the average of and difference between velocities signaled by visual and vestibular inputs (*SI Appendix*, Fig. S1 *A*, *Bottom*). Fusion and scission unit estimates represent (integrated) self- and scene motion, respectively. For self-motion, we considered the case when cues are integrated, because self-motion is derived from the unisensory vestibular signal when cues are separated. Assessing performance against the training objectives, we found a high correspondence (all *r* > 0.95) between the actual and estimated self- and scene-motion velocities, and causal inference (i.e., integrate versus separate) was achieved with ∼85% accuracy across movement directions (chance = 50%). We used a range of training regimes to ensure that our findings generalized across different stimulus distributions (*SI Appendix*, Fig. S1 *B* and *C*). We then explored the network’s behavior in detail to uncover the computations that underlie causal inference.

### Behavioral Phenomena.

**Fig. 2.**
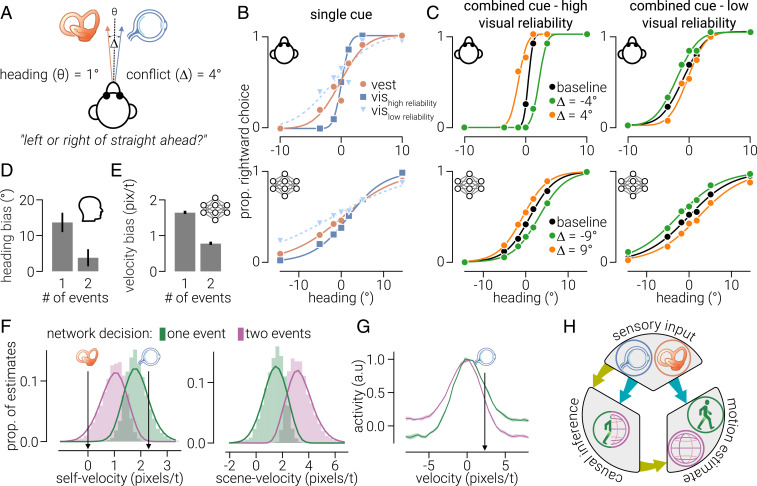
Reliability-based cue weighting and relationship between causal inference and motion judgements. (*A*) Macaques were presented with vestibular (moving platform) and visual cues (dot motion) signaling forward movement at a range of offsets around straight forward. A small cue conflict was introduced by displacing cues (±4°) in opposite directions from the test offset. The macaques’ task was to indicate whether the direction of movement was to the left or right of straight ahead. (*B*, *Top*) Single-cue performance on the task using just vestibular (brown dots) cues and visual cues with high (dark blue squares) or low (light blue triangles) reliability. (*C*, *Top*) Performance on the task using combined cues with no conflict (black), −4° (green), or 4° (orange) conflict when the reliability of the visual cue is high (*C*, *Top Left*) or low (*C*, *Top Right*). (*B* and *C*, *Bottom*) Same as *B* and *C*, *Top* but from self-motion estimates decoded from MultiNet; note, for the network we used a cue conflict of ±9°. Data in *B* and *C*, *Top* extracted and replotted from ref. 9; the illustration shown in *A* is also adapted from (9). (*D*) Data from ref. 18 showing human self-motion judgement biases of dot motion stimuli consistent with forward motion containing a localized region in which dots move to the right. Average biases are shown for trials on which participants judged there was just global motion (one event) or an additional local motion cue (two events). Error bars indicate SEM. (*E*) Same as *D* but for MultiNet self-motion judgements of visual and vestibular cues on the decision boundary of causal inference. Error bars indicate bootstrapped 95% CIs. (*F*) Distribution of self- (*Left*) and scene-motion (*Right*) MultiNet estimates for trials on which the network judged there to be either one or two events. Solid lines indicate rolling averages across five histogram bins, and arrows indicate the velocities of vestibular and visual inputs. (*G*) The average vestibular input across trials that the network judged there was either one (green) or two (magenta) events. Shaded regions indicate 95% CIs. (*H*) Illustration of possible information flow resulting in biased judgements. The decision of causal inference, based on sensory input, may influence judgements of self-/scene motion (yellow arrows). Alternatively, both causal inference and self-/scene-motion judgements may be decoded from sensory input (cyan arrows). The latter is how MultiNet operates (i.e., self-/scene motion and causal inference are decoded from MSTd activity). To implement the former process, the self-/scene-motion units in MultiNet could either be placed after the causal inference units or be influenced by them via lateral/feedback connections.

We first assessed a key aspect of perceptual integration: when human and nonhuman primates combine multisensory signals, they weight them according to the reliability of each (9, 17). In particular, if a small conflict is introduced between two cues, an observer’s judgment will incorporate both signals but depend more on the signal that is more reliable (i.e., has the lowest estimator variance) (9, 10, 21). Consider an observer judging the direction of movement when visual and vestibular cues indicate slightly different directions (Fig. 2*A*). If the visual cue (depicted in a random-dot pattern) is more reliable, the observer’s direction judgment is closer to the signal provided by the visual cue (Fig. 2 *C*, *Top Left*). However, experimentally manipulating the cues to make the visual cue less reliable (e.g., by reducing the motion coherence of the pattern) results in judgments that are closer to the vestibular cue (Fig. 2 *C*, *Top Right*) (9). When we used this paradigm to test MultiNet, we found that it recapitulated reliability-based cue weighting: when a small conflict was introduced between vestibular and visual inputs, the network’s integration unit estimates were biased toward the more reliable cue (Fig. 2 *B* and *C*, *Bottom*).

The reweighting of cues according to their reliability has been observed in individual MSTd neurons (*SI Appendix*, Fig. S2 *A* and *B*, *Top*) (9); the neuronal tuning curves shift up or down depending on both the cue reliability and direction of conflict. Neurons respond to cue conflict with a change in firing rate depending on which cue is more reliable. If a neuron prefers leftward heading angles, when the visual cue is more reliable, this results in higher firing rate when the visual cue is offset to the left relative to baseline (and vice versa for rightward offsets; *SI Appendix*, Fig. S2 *B*, *Top Left*). By contrast, when the visual cue is less reliable, the direction of the shift is reversed (*SI Appendix*, Fig. S2 *B*, *Top Right*). We tested MultiNet and found the same mechanism present among the MSTd congruent units (*SI Appendix*, Fig. S2 *A* and *B*, *Bottom*). We further found that the precision of MultiNet’s motion estimates matched those measured psychophysically on the task and were consistent with predictions of optimal integration (*SI Appendix*, Fig. S2 *C* and *D*).

Next, we considered the relationship between perceptual judgments and how we attribute sensory signals to events in the environment (i.e., whether we decide that two signals were caused by the same or different events). Dokka et al. (18) asked participants to judge leftward versus rightward looming motion in optic flow patterns, in which an additional local patch of left versus right motion was superimposed. Observers judged both motion direction and whether there had been additional motion (two events) or not (one event). Observers’ judgements were more biased in the direction of the local motion on trials in which they reported only one event (Fig. 2*D*), suggesting that when both cues were attributed to the same cause they were integrated, but they were separated when two causes were detected by the observers.

We performed a conceptually similar experiment with the network, using stimuli that MultiNet was trained for (i.e., we did not examine local versus global motion because it was not trained on these stimuli). In Dokka et al. (18), the two signals that could be attributed to the same or different events were a localized patch of motion moving to the right within a larger field of optic flow signaling forward motion. We replaced these signals with vestibular and visual input signaling different velocities along the *x*-axis. We found the same pattern of results (Fig. 2*E*): left/right directional velocity estimates were more biased by the visual input on trials in which both signals were attributed to one event. This bias is equivalent to the illusion of self-motion that can occur when viewing a moving train from one that is stationary; that is, errors in causal inference can occur when sensory cues caused by different events are similar. Additionally, we found the opposite effect for scene-motion judgements: directional velocity estimates were less biased toward the vestibular input on trials in which signals were attributed to two events (Fig. 2*F*).

Dokka et al. (18) suggest that the act of causal inference subsequently biases perceptual judgements (Fig. 2*H*, yellow arrows). This cannot occur in the feedforward MultiNet architecture, yet the bias persists. To understand why, we examined the vestibular signals that gave rise to different causal inferences (Fig. 2*G*). Because of simulated internal noise at the vestibular input stage (*Methods*), on approximately half the trials, the vestibular signal was shifted away from zero toward the visual signal. This shift in vestibular activity gave rise to both the inference of one event and self-motion estimates biased toward the scene-motion (visual) event. Thus, the relationship between causal inference and perceptual judgements can be explained by the same underlying activity (Fig. 2*H*, cyan arrows).

### Multisensory Tuning Properties.

**Fig. 3.**
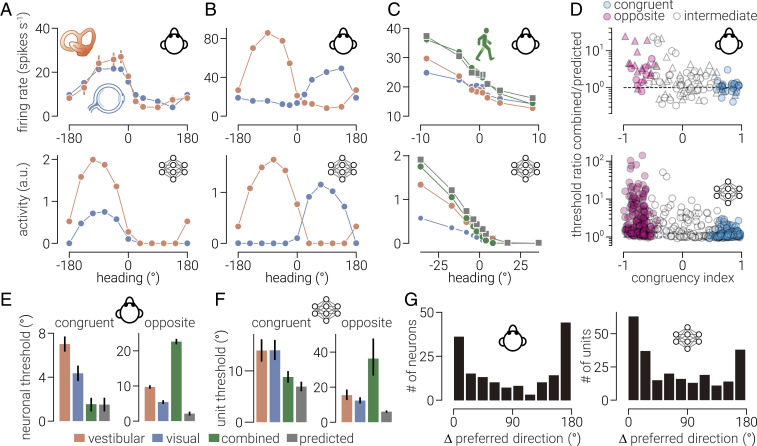
Multisensory tuning properties of MultiNet MSTd units. (*A* and *B*, *Top*) The responses of two macaque MSTd neurons to visual (blue) and vestibular (orange) cues as a function of heading direction. The tuning of one neuron is the same for both cues (*A*, congruent neuron), while the tuning of the other neuron is different (*B*, opposite neuron). (*C*, *Top*) Same as *A*, *Top* but for a narrower range of heading directions around straight forward, including the response to both cues at the same time (green) and the response predicted by a linear summation model (gray). (*D*, *Top*) The ratio between the neuronal threshold of macaque MSTd neurons for congruent visual and vestibular cues to that predicted by optimal cue integration as a function of the congruency between their tuning for these cues in isolation. Filled symbols denote neurons for which the congruency index significantly differs from zero. Triangle and circles denote data from different monkeys. The bottom of *A* through *D* shows the same analysis as the top but for MultiNet MSTd units. (*E*) Average neuronal thresholds for (left) congruent and (right) incongruent neurons. Error bars indicate SEM. (*F*) Same as *E* but for MultiNet MSTd units. (*G*, *Left*) The distribution of macaque MSTd neurons as a function of the difference between preferred visual and vestibular heading direction. (*G*, *Right*) Same as *G*, *Left* but for MultiNet MSTd units. Data shown in *A* through *D*, *Top* and *E* were extracted from ref. 8, and data shown in *G*, *Left* were extracted from 6. Error bars in *E* and *F* indicate SEM between neurons and bootstrapped 95% CIs, respectively.

Macaque MSTd neurons sensitive to both visual and vestibular signals tend to prefer either the same (i.e., congruent neurons; Fig. 3 *A*, *Top*) or opposite directions (i.e., opposite neurons; Fig. 3 *B*, *Top*) within the two modalities (8). Gu et al. (8) quantified the sensitivity of MSTd neurons to congruent combinations of visual and vestibular cues by decoding (left/right) direction from their response profiles (Fig. 3 *C*, *Top*). They found that the thresholds of congruent neurons were similar to those predicted by optimal cue integration, whereas the thresholds of opposite neurons were considerably higher (Fig. 3 *D*, *Top* and *E*). We performed this experiment on MultiNet and found the same pattern of results: MSTd units preferred either congruent or opposite visual–vestibular directions (Fig. 3 *A* and *B*, *Bottom*), and the thresholds of congruent but not opposite units were near optimal (Fig. 3 *C* and *D*, *Bottom* and *F*).

Moreover, the distribution of differences between the network’s MSTd units’ visual–vestibular preferred direction was similar to that found in macaque MSTd (Fig. 3*G*) (6) (i.e., bimodal with peaks at 0° and 180°). This is consistent with our previous modeling work, where we found that robust cue integration of depth cues can be achieved using cue preferences that are bimodally clustered around congruent and opposite (22). We evaluated MultiNet’s properties after different numbers of training epochs, and found that these properties emerge early during training and then remain stable (*SI Appendix*, Fig. S3). Further, we found that the same properties emerge in a network trained on a uniform distribution of heading directions and speeds (*SI Appendix*, Fig. S4).

### Solving Causal Inference.

**Fig. 4.**
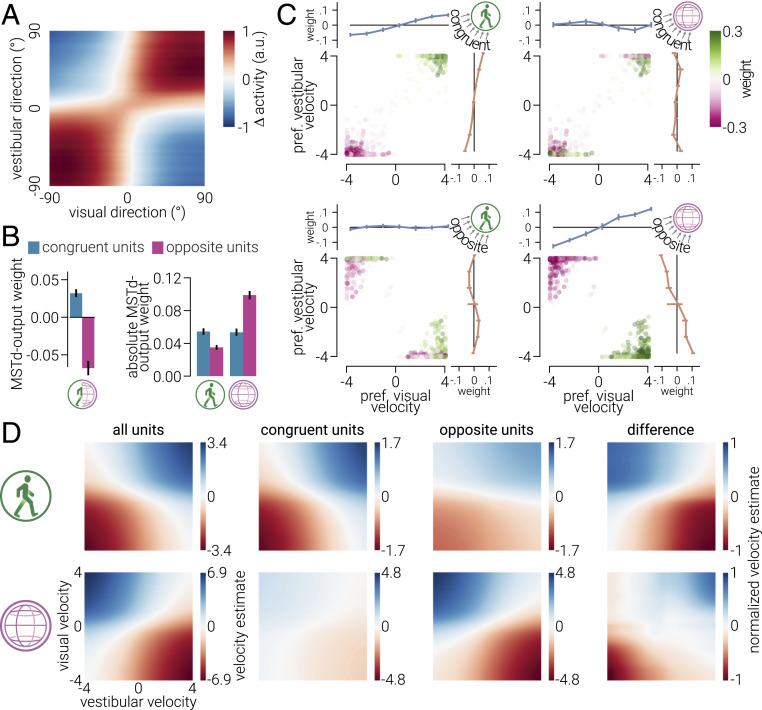
Solving causal inference with an artificial network. (*A*) The difference between the average response of congruent and opposite MultiNet MSTd units to different combinations of visual and vestibular signals. Positive (red) values indicate congruent units are more active, and negative (blue) indicate opposite units are more active. Note that activity is weaker at the center (around 0° direction; straight ahead motion), because the congruent/opposite classification method is not designed to categorize units tuned to near-zero directions. (*B*, *Left*) The average weights between congruent and opposite MSTd units and the causal inference decision units. (*B*, *Right*) The same as *B*, *Left* but for absolute weights between MSTd units and fusion/scission regression units. (*C*) The weights between (opposite/congruent) MSTd units and (fusion/scission) regression units as a function of their preferred visual and vestibular velocity. The average weights, binned as a function of the velocity to which MSTd units were maximally responsive, are shown above and to the right of scatter plots. Error bars in *B* and *C* indicate bootstrapped 95% CIs. (*D*, *Top*) Self- and (*Bottom*) scene-velocity estimated by the network in response to a range of visual–vestibular inputs decoded from the activity of all the MSTd units, the congruent units, or the opposite units. The final column shows the difference in (normalized) velocity estimates from congruent and opposite units (opposite minus congruent). All visual velocities are in pixels/frame and vestibular velocities are equivalently mapped to visual velocity; weights are in arbitrary units (a.u.).

To understand how congruent and opposite units compute causal inference, we measured the responses of these subpopulations to different combinations of visual–vestibular input. As expected, congruent and opposite units were more active in response to similar and dissimilar combinations of these cues, respectively (Fig. 4*A*). We reasoned that the relative activity of these populations could be used to solve causal inference; that is, when congruent units are more active, signals are attributed to the same event, and when opposite units are more active, they are attributed to different events. In line with our reasoning, we found that congruent and opposite units primarily promoted decisions to combine and separate cues, respectively (Fig. 4 *B*, *Left*).

We next examined how motion estimates are decoded from the activity of congruent and opposite units. We found that congruent units primarily determined self-motion estimates while opposite units primarily determine scene-motion estimates (Fig. 4 *B*, *Right*). The former is consistent with neurophysiological findings (6), while the latter explains the role of previously puzzling opposite neurons. Despite congruent and opposite units showing primacy for particular tasks, we found that they both contributed to self- and scene-motion estimates. To understand how, we examined the weights connecting congruent/opposite MSTd units to fusion/scission output units as a function of the preferred visual and vestibular direction of the former (Fig. 4*C*). Congruent-fusion connections promoted the velocities to which they were tuned. Opposite-scission connections were similar but inverted between preferred cue velocity. While congruent-fusion and opposite-scission connections were linearly related to preferred cue velocity, congruent-scission and opposite-fusion connections adhered to a sinusoidal pattern. The sinusoidal shape represents a slight bias of the network for smaller velocities. The cost function associated with these weights is the difference between the real and predicted velocity. Thus, the bias is likely a strategy for reducing error under conditions of uncertainty (i.e., the most rational estimate to reduce the difference between the real and predicted number is the center of the distribution). Further, the sinusoidal pattern of congruent-scission rather than opposite-fusion connections was inverted between preferred cue velocity. The similarity of congruent-fusion connections and inversion of opposite-scission connections makes intuitive sense because it matches the tuning of the MSTd units (e.g., congruent MSTd units are tuned to similar directions between cues so we would expect to see similar visual and vestibular connections). Thus, it is surprising that this relationship is reversed for congruent-scission and opposite-fusion connections (i.e., congruent-scission connections are inverted while opposite-fusion connections are similar). This suggests that the congruent units partially act as opposite units and vice versa.

To causally interrogate how congruent and opposite units support decoding self- and scene motion, we measured velocity estimates after artificially lesioning these unit types (Fig. 4*D*). We found that when self-motion is decoded from the activity of opposite units, the pattern of estimates is similar to that decoded from congruent (or all) units. Similarly, scene-motion estimates decoded from congruent units were qualitatively similar to those from opposite (or all) units. However, in both cases, the scale of estimates was considerably lower, which is consistent with their lesser role in influencing these outputs. We then measured the difference between the normalized estimates decoded from opposite and congruent units and found that the difference matched the pattern of estimates for the units’ primary connections. These results show that congruent and opposite units can serve each other’s computational roles to an extent, but they continue to exhibit behavior consistent with their primary function. How is this possible? One explanation is that this is achieved by small differences in preferred cue direction (e.g., a congruent unit with a small separation between preferred cue directions may act as an opposite unit). If this were the case, we would expect a positive correlation between the difference in cue velocity to which congruent units are tuned and the extent of their influence on scene-motion estimation. By contrast, we found a negative correlation between these factors (*r* = −0.19, CI_95%_ = [−0.26, −0.13]). An alternative explanation is that the dual roles are achieved through the units’ asymmetric cue sensitivity. For example, an MSTd unit may prefer opposite cue directions while also being more sensitive to one of those cues. In response to congruent cues of similar reliability, the unit’s response may approximate unisensory or congruent rather than opposite selectivity (*SI Appendix*, Fig. S5). In line with this explanation, we found a positive correlation between cue sensitivity asymmetry and magnitude of congruent-scission (*r* = 0.54, CI_95%_ = [0.48, 0.60]) and opposite-fusion weights (*r* = 0.39, CI_95%_ = [0.34, 0.45]).

## Discussion

Judging whether independent sensory signals were produced by the same or different events is a fundamental challenge faced by biological systems. Several neural loci of cue integration have been studied extensively; however, the computational mechanisms that instantiate causal inference remain elusive. Here, we trained an artificial system to perform causal inference within the context of combining visual and vestibular signals to estimate self- and scene motion. The network learns the task, and in the process, it develops properties that match those found in biological systems at neural and behavioral levels. We use our unrestricted access to the artificial system to explain the relationship between causal inference and motion judgements and how congruent and opposite units solve causal inference while simultaneously computing estimates of self- and scene motion.

Self-motion can be inferred from global patterns of visual motion. When the (local) motion of an independently moving object is sufficiently similar to this pattern of global motion, human estimates of self-motion are more biased toward the local motion cue when they are both attributed to the same event (18). This may be due to feedback, triggered by the causal inference decision, that alters the representation of the cues. However, we found the same effect in the network, which is exclusively feedforward, when we performed a conceptually similar experiment using visual and vestibular cues (rather than global and local visual motion cues). Although the same visual–vestibular difference is used on each trial, the noise added to the signals results in approximately equal numbers of trials on which they are attributed to the same or different events. This is equivalent to the human experiment, in which there were both internal (e.g., variable neuronal resting potential) and external sources of noise (e.g., variability in the visual stimulus). We showed that on trials in which the noise of the primary (vestibular) signal shifted its population activity toward the velocity represented by the secondary (visual) signal, the probability that the two signals would be combined was increased, as their difference was reduced. The same shift in activity that increased the probability of cue combination produced estimates that were more biased toward the second cue. Thus, the relationship between causal inference and motion judgements can be explained by the same underlying activity rather than a serial process from one to the other (i.e., feedback).

Although we demonstrated this phenomenon using simulated visual and vestibular cues, it seems reasonable that the same principle can also be applied to combining two visual cues (e.g., global and local motion). Future experimental work could test this idea. For example, the likelihood of attributing both signals to the same event could be recalibrated through repeated exposure (22–25) without changing the underlying signals. This would be equivalent to manipulating the weights between the MSTd layer and the causal inference units in MultiNet. If the act of causal inference subsequently biases motion estimates, this should produce more biased estimates. However, if causal inference and motion estimates are explained by the same underlying activity, this manipulation should not affect motion estimates. The visual input used to train and test MultiNet comprised whole field contributions of self- and scene motion. Scene motion can also be spatially localized within the visual field, similar to the stimuli used by Dokka et al. (18) (i.e., object-motion). Future work could investigate the computations that emerge in a similar network to parse spatially localized scene motion from self-motion.

The differences between biological and artificial neural systems far outweigh their similarities: we are considering only a very small portion of one sensory processing hierarchy that is optimized for a single and specific task without meaningful constraints on the energy required to perform the computations and artificial neurons that are radically simpler than biological neurons. Despite these wide differences, we found that MultiNet developed congruent and opposite units like those in macaque MSTd. From the balance in activity between these unit type subpopulations, the network determined whether multisensory signals arose from the same or different events. That is, when congruent units are more active, cues are combined, whereas when opposite units are more active, cues are separated. This mechanism could be implemented within biological systems through mutual suppression between the two subpopulations. We defined a decision boundary (0.5) for the activity of MultiNet’s causal inference units, which determined inference selection. However, this activity (between 0 and 1) could also be used to weight the alternatives, such as is performed by theoretical Bayesian models of causal inference (26–31). This suggests that the activity in multisensory loci, such as MSTd, is sufficient to perform causal inference without high-level cognitive involvement.

We explored how self- and scene-motion estimates are decoded from the activity of congruent and opposite units and found that congruent units primarily determined self-motion estimates while opposite units primarily determine scene-motion estimates. The former is consistent with previous neurophysiological work (6), whereas the latter provides an explanation for the existence of opposite neurons. Despite congruent and opposite units showing primacy for particular tasks, we found that they both contributed to both motion estimates. We found that this was made possible by asymmetries in sensitivity to different cues, which are also common among macaque MSTd neurons (6, 8, 10, 11).

These findings can guide future experimental work. Given the wide repertoire of known biological properties that emerged within MultiNet, it seems reasonable that our simplified network provides a first approximation of the computational processes used by biological brains to perform causal inference and motion estimation. Previous theoretical modeling work has indicated that opposite neurons in macaque MSTd may be involved in segregating sensory cues (22, 32, 33). However, the approach used here provides a complete end-to-end artificial system which receives simulated vestibular and visual inputs to produce readout “perceptual” estimates. It is important to note that the neural network was not constrained to reproduce known biological properties; rather, these properties emerged through training from biologically plausible input and task structures.

We have considered the problem of causal inference in the context of combining visual and vestibular cues (30, 34). While this domain has provided specific insight into congruent and opposite representations for multimodal motion cues, there are other cases where similar tuning properties emerge (e.g., binocular disparity and motion parallax cues) (35, 36). The abstracted nature of the vestibular signals presented to MultiNet leads us to think that this functional architecture may present a canonical solution for computing the integration versus separation of different sensory estimates. However, test cases involving other sensory pairings may prove informative to test the generality of the network solutions we have uncovered (e.g., audiovisual cues to spatial location or more challengingly, taste–color pairings).

Recent advances in machine learning have produced deep neural networks comprising many layers that surpass human performance on many tasks (e.g., object recognition) (37, 38). However, understanding of their internal processes is often as limited as in biological systems due to their scale and complexity. Here, we constrain the size of the artificial system, allowing us to apply in silico measurement techniques that lay bare the processes that underlie multisensory integration, separation, and causal inference. We demonstrate how optimizing motion estimation in an artificial network using natural images and simulated vestibular signals recapitulates a range of neurophysiological and perceptual phenomena. It allows us to explain how puzzling properties of biological neurons can be used to compute causal inference within a relatively simple feedforward network, without the need for high-level top-down feedback.

## Methods

### Naturalistic Motion Sequences.

We generated motion sequences using photographs of the natural world (39). Images were greyscale outdoor scenes (converted from red-green-blue using MATLAB’s [The MathWorks, Inc., Matick, MA] *rgb2grey* function). Motion sequences (six frames) were produced by translating, zooming, and rotating a 64 × 64 pixel cropped patch of the image (Fig. 1*A*). Image velocity was randomly assigned from a uniform distribution between ±4 pixels/frame, separately in four dimensions (*x*, *y*, *z*, and rotation [*r*]). These axes of motion were selected (i.e., rather than yaw and pitch) based on previous neurophysiological evidence of neuronal selectivity (40) and to reduce the complexity of the spatiotemporal image manipulations required to generate the visual stimuli. Motion-in-depth and rotation of the observer produce image speeds that increase as a function of distance from the focal point, whereas translational motion produces uniform image speed; thus, to equate the image speeds between velocity dimensions we selected a range of motion-in-depth and rotation speeds that produced the same speed at the midpoint between the focal point (center of the image) and the edge of the image as translational speed.

To produce motion-in-depth (*z*), image sequences were initiated at a resolution that was down-sampled from the original, to avoid blurring due to low image resolution at close distances, then either up- or down-sampled on subsequent frames. Starting at a down-sampled resolution allowed subsequent up-sampling without creating new pixels values. This method produced a pattern of expanding/contracting radial motion centered on a focal point that was determined by the translational image velocity. Motion-in-depth and rotational motion produced nonuniform motion speeds across the image that increased with distance from the focal point; thus, the randomly assigned velocity corresponded to the motion speed at a location half-way between the focal point and the edge of the image when the focal point was centered on the image. Image translation, motion-in-depth, and rotation were performed in MATAB using Psychtoolbox version 3.0.11 subpixel rendering extensions (41, 42) (http://psychtoolbox.org/). The speeds used to train the network were selected because they did not exceed the image dimensions (64 × 64 pixels) and were similar to those used in our previous studies (15, 16). We generated 64,000 motion sequences, which were scaled so that pixel intensities were between –1 and 1 and randomly divided into training and test sets, as described in the *Training Procedure* section.

### Network Architecture.

All the networks described in the study were implemented in Python version 3.6.4 (https://python.org) using TensorFlow (http://www.tensorflow.org), a library for efficient optimization of mathematical expressions. We used a convolutional neural network that comprised two streams of input that represented visual and vestibular sensory signals (Fig. 1*B*). The visual input stream comprised an input layer, one convolutional-pooling layer, and one fully connected layer. The vestibular stream comprised an input layer and one fully connected layer. The two streams converged on a fully connected layer, which was read out by an output layer comprising eight regression units and four binary units.

Visual stream inputs were image patches (64 × 64 × 6 pixels; the last dimension indexing the motion frames). In the convolutional layer, inputs passed through 64 three-dimensional kernels (12 × 12 × 6 pixels), producing 64 two-dimensional output maps (53 × 53 pixels). This resulted in 179,776 units (64 maps of 53 × 53 pixels) forming 155,326,464 connections to the input layer (64 maps of 53 × 53 × 12 × 12 × 6 pixels). Since mapping is convolutional, this required that 55,360 parameters were learned for this layer (64 filters of dimensions 12 × 12 × 6 plus 64 offset terms). We chose units with rectified linear activation functions to model neurophysiological data (43). The activity, *a*, of unit *j* in the *k*th convolutional map was given by:

$$a_{j}^{\left(k\right)}=w^{\left(k\right)}s_{j}+b_{j}^{\left(k\right)},$$ [1]

where *w*^(*k*)^ is the 12 × 12 × 6 three-dimensional kernel of the *k*th convolutional map, *s*_j_ is the 12 × 12 × 6 motion sequence captured by the *j*th unit, and *b*_j_ is an offset term. Parameterizing the motion image frames separately, the activity $a_{j}^{\left(k\right)}$ can be alternatively written as:

$$a_{j}^{\left(k\right)}=\left(\sum w^{\left(t_{n}k\right)}s_{j}^{t_{n}}\right)+b_{j}^{\left(k\right)},$$ [2]

where $w^{\left(t_{n}k\right)}$ represent the *k*th kernels applied to motion image frames (i.e., receptive fields at times 1 to 6), while $s_{j}^{t_{n}}$ represent the input images captured by the receptive field of unit *j*.

A fully connected layer (2,985,984 connections; 46,656 per feature map, resulting in 2,986,048 parameters including the 64 offset terms) mapped the activities in the pooling layer to 64 fully connected units. All fully connected layer activities *r* were obtained by mapping the vector of activities in the preceding layer a via the weight matrix *W* and adding the offset terms *b*:

r=Wa+b. [3]

Vestibular stream input comprised four Gaussian distributions, one for each velocity dimension, produced with the following:

$$y\left(x\right)=\text{exp}\left(\frac{{\left(x-\mu \right)}^{2}}{-2\sigma^{2}}\right),$$ [4]

where *x* assumed 32 evenly spaced values between ±8, *μ* denotes the velocity, and the SD (*σ*) was randomly selected from a uniform distribution between 1 and 8. Note, biological vestibular systems are sensitive to acceleration, not constant motion; thus, while for simplicity, we define inputs by velocity, they represent acceleration from inertia to movement. The vestibular stream input thus consisted of 128 values (32 × 4). Gaussian noise (mean = 0, SD = 0.3) was added to the vestibular input to prevent the network from using only the maximum value within each distribution to perform the task. A fully connected layer (1,536 connections; 128 per unit, resulting in 1,548 parameters including the 12 offset terms) mapped the activities of the vestibular input layer to 12 fully connected units.

A fully connected layer (4,864 connections; 4,096 visual stream connections and 768 vestibular stream connections, resulting in 4,940 parameters including the 64 visual and 12 vestibular offset terms) mapped activities from the visual and vestibular streams to 64 fully connected units. Finally, an output layer (768 connections, 64 for each of the eight regression and four binary units, resulting in 832 parameters including the eight offset terms) mapped activities from the fully connected layer to eight regression units, which represented the average- and difference-velocity solutions along the four (*x*, *y*, *z*, and *r*) dimensions, and four binary units, which represented the decision of whether the visual and vestibular signals were produced by a single event or two events. The regression unit activities were obtained using Eq. **3**. The binary unit activities were obtained by applying a sigmoidal activation function

$$y=\frac{1}{\left(1+\text{exp}\left(-r\right)\right)},$$ [5]

to the result of Eq. **3**. A rectified linear activation function was applied to the activation of all layers prior to the output units.

### Training Procedure.

Visual and vestibular input pairs were randomly divided into training (75%, *n* = 48,000) and test (25%, *n* = 16,000) sets. No pairs were simultaneously present in the training and test sets. To optimize the network, only the training set was used. We initialized the weights of the convolutional layer as Gaussian noise (mean, 0; SD, 0.001). The weights in the fully connected and regression layers and all offset terms were initialized to zero.

The network was trained using minibatch gradient descent with each batch comprising 32 randomly selected examples. For each batch, we computed the derivative of the loss function with respect to parameters of the network via back-propagation and adjusted the parameters for the next iteration accorded to the update rule:

$$w_{i+1}=w_{i}-\alpha \langle \frac{\partial L}{\partial w_{\left(D_{i}\right)}}\rangle ,$$ [6]

where *α* is the learning rate and $\langle \frac{\partial L}{\partial w_{\left(Di\right)}}\rangle$ is the average over the batch *D*_*i*_ of the derivative of the loss function with respect to the *w*, evaluated at *w*_*i*_. The learning rate *α* was constant and equal to 1.0 × 10^−4^. After evaluating all the batches once (i.e., completing one epoch), we tested the network using the test image dataset. We repeated this for 50 epochs. The loss of the regression units was calculated as the mean squared error, and the loss of the binary units was calculated as binary cross-entropy. Loss weights of 1.0 and 0.2 were used for the regression and binary outputs, respectively, as it was found that the network failed to reach sufficient estimation accuracy when equal weights were used.

For each of the four velocity dimensions, there were three output units: two regression units, and one binary unit. The fusion and separation regression units were to estimate self- and scene velocity, which were calculated as the average and difference between vestibular and visual velocities, respectively. The range of scene-velocity values was twice that of self-velocity values. Thus, to prevent scene-velocity estimation from dominating the training process, a custom loss function was used to equate the potential loss associated with the two estimates by halving the loss associated with the scene-velocity estimate. The binary units were tasked with detecting whether the difference between visual and vestibular signals was larger than the median absolute difference across all 64,000 pairs (∼2.3). Although the binary units produced sigmoidal activation, they were trained on a binary distribution of solutions. Thus, for all analyses, we interpreted their output as binary (i.e., above or below 0.5).

To test the effect of using nonuniform distributions of self- and scene-motion labels during training, we trained separate (self-/scene-motion) control networks on uniform distributions of these solutions. We create training sets with uniform distributions self- or scene-motion solutions by using nonuniform distributions of visual and vestibular input data.

### Generation of Test Stimuli.

Dot motion stimuli were used to test the response of the network after it had been trained on natural images. These were generated with in-house scripts in Python. Unless otherwise specified, these stimuli comprised 50 randomly positioned dots that moved at 4 pixels/frame. Dots were either black (pixel value, −1) or white (pixel value, 1), selected at random, two-dimensional Gaussians (diameter, 6; SD, 1.2) on a midgray background (pixel value, 0). When dots moved beyond the image border, they were repositioned at a randomly selected location. For radial and rotational motion, dot speed varied linearly as a function of distance from the focal point such that dots midway between the focal point and the edge moved at half the specified speed as dots at the edge. For negative radial motion (i.e., motion toward the focal point), dots that reached the focal point of the image were repositioned to a random location.

### Demonstration of Replicability.

To demonstrate the consistency of training outcomes, five networks were trained using the same conditions, and the results shown are either the average or sum of the networks, and error bars indicate variability between networks. Similarly, to demonstrate consistency across stimuli, which had randomized initiation properties (e.g., dot position) the results shown are the average of 64 visual and vestibular input stimuli.

### Statistical Analyses.

To assess the significance of differences between parameters, bootstrapping (50,000 iterations) was used to calculate 95% CIs (CI_95%_). The Pearson’s correlation was used to assess the significance of relationships between parameters.

### Decoding Direction and Speed.

Where direction and/or speed are reported in polar coordinates, these estimates were produced by converting *x* and *y* or *z* and *r* velocity estimates to speed *ρ* and direction *ϕ* with the following:

$$\rho =\sqrt{v_{x}^{2}+v_{y}^{2}}$$ [7]

$$\phi =\text{arctan}2\left(v_{x},v_{y}\right),$$ [8]

where *v*_*x*_ and *v*_*y*_ denote *x* and *y* or *z* and *r* velocity vectors (*SI Appendix*, Fig. S6).

### Reliability-Based Cue Weighting.

To compare the reliability-based cue weighting of the network to psychophysical recordings from macaque (extracted and replotted psychophysical data from figure 1 of ref. 9), we measured the network’s self-motion estimates of conflicting visual and vestibular input at different levels of reliability. In line with ref. 9, we combined motion-in-depth and velocity along the *x* dimension to produce “heading direction” to the left and right (along the x dimension) of straight forward (seven evenly spaced directions between ±14°). Two motion coherence levels, high (100%) and low (60%), and three conflict conditions (0 and ±9°) were tested. In conflict conditions, visual and vestibular cues were each offset in opposite directions at the above-stated angle such that their average angle was equal to the test angle. The vestibular signal (SD, 6) included Gaussian noise (mean, 0; SD, 0.3). 128 trials were run for each test direction, and the proportion of rightward estimates as a function of test direction was fit with a sigmoid function.

### Causal Inference Decision Bias.

To compare the estimation bias for different causal inference decisions of the network to psychophysical recordings from humans (extracted and replotted psychophysical data from figure 4 of ref. 18), we measured the network’s self- and scene-motion estimates to incongruent visual and vestibular input, separated by the velocity difference (2.3) that defined the border between fusion and scission for decision units of the network during training. Vestibular input (SD, 2) was set at 0 velocity with Gaussian noise (mean, 0; SD, 0.8) added, and visual input was set at either ±2.3 velocity. Tests were performed within the *x* velocity dimension. In total, 2,560 trials were performed for each velocity separation, and self- and scene-motion estimates were grouped by the decision made by the network regarding the number of events that produced the signals (1 or 2). The velocity separation of signals was at the decision border; thus, on approximately half of the trials, the network decided there were two events. The average self-motion was calculated for each decision-based distribution.

### Multisensory Tuning Properties.

To compare the tuning properties of the network to those of neurons in macaque dorsal MSTd area (extracted and replotted neurophysiological data from figures 2A and D and 3 of ref. 8 and figure 6D of ref. 6), we measured the network’s response to visual and vestibular input in isolation and in (congruent) combination. In single-cue conditions, the excluded cue was input as zeros. In line with previous work (6, 8), we combined motion-in-depth and velocity along the *x* dimension to produce “heading direction.” To establish the MSTd units’ tuning functions, we measured their response to visual and vestibular input in isolation in 10 evenly spaced heading directions from 0 to 360°, where 0° represents straight ahead motion-in-depth and 90° represents motion directly to the right. We then fit sinusoidal functions to these responses to determine their preferred visual and vestibular heading direction:

$$y=a\times \text{sin}\left(x-x_{\text{pref}}\right)+b.$$ [9]

Consistent with previous work (6), only units which were considered to have strong tuning (fitted amplitude [*α*] parameter > median across all units) were included in the histogram showing the number of units as a function of the difference between preferred visual and vestibular direction (*x*_pref_).

To test the MSTd units’ sensitivity to changes in heading direction, we measured their response to visual and vestibular input in isolation and in congruent combination to 10 logarithmically space directions between ±36°. To characterize the units’ sensitivity from these data, we used receiver operating characteristic (ROC) analysis to calculate the ability of an ideal observer to discriminate between two opposite-directed headings (for example, +36° versus −36°) based on the response of the unit and a presumed anti-unit with opposite tuning (44). We then constructed synthetic–neurometric functions from these ROC values and fitted these with sigmoid functions to determine unit thresholds. We calculated the predicted unit sensitivity for combined input, assuming maximum likelihood cue integration with the following:

$$\sigma_{\text{predicted}}=\sqrt{\frac{\sigma_{\text{vesibular}}^{2}\sigma_{\text{visual}}^{2}}{\sigma_{\text{vesibular}}^{2}+\sigma_{\text{visual}}^{2}}},$$ [10]

where *σ*_vesibular_ and *σ*_visual_ denote the MSTd units’ threshold for change in heading direction as indicated by vestibular and visual input, respectively.

Consistent with previous work (8), we quantified the congruency between the visual and vestibular tuning functions of the MSTd units measured during discrimination by calculating their congruency index. A Pearson correlation coefficient was first calculated for each single-cue condition. This quantified the strength of the linear trend between firing rate and heading for vestibular (*r*_vestibular_) and visual (*r*_visual_) stimuli. Congruency index was defined as the product of these two correlation coefficients:

$$\text{CI}=r_{\text{vestibular}}\times r_{\text{visual}}.$$ [11]

The congruency index ranges between ±1, with values near 1 indicating that visual and vestibular tuning functions have a consistent slope and values near −1 indicating opposite slopes. This congruency index reflects both the congruency of tuning and the steepness of the slopes of the tuning curves around straight ahead (0°). We considered the congruency index to be significantly different from 0 when both of the constituent *r* values were significant (*P* < 0.05). We denoted units having values of congruency index significantly different from 0 as congruent (congruency index > 0) or opposite (congruency index < 0). Units with nonsignificant congruency indices were considered intermediate.

### Causal Inference.

To understand how congruent and opposite MSTd units contribute to the decision to integrate or separate visual and vestibular input, we measured the difference in response of opposite and congruent MSTd units to all combinations of visual and vestibular cues between ±90° heading direction (35 evenly spaced intervals). To further explore this relationship, we next categorized all MSTd units as either congruent or opposite for each velocity dimension in isolation, rather than heading direction, by calculating the Pearson *r* coefficient between their response to visual and vestibular input at seven evenly spaced speeds between ±4 pixels/frame. The coefficient was used in the same manner as the congruency index to categorize units as either congruent, opposite, or intermediate. To assess the roles of congruent and opposite units on the decision of the network, we calculated the average weights between these units and the decision nodes. We found the same pattern of results for all velocity dimensions; thus, we collapsed the results across dimensions.

To test whether congruent and opposite MSTd units have different levels of influence on the estimation of self- and scene velocity, we computed the average absolute weights between these units and the regression output units. Velocities can be either positive or negative. Thus, positive and negative weights between MSTd and regression units do not reflect excitation and inhibition but rather the direction and magnitude in which the MSTd unit’s activity drives the velocity estimation. The absolute weight provides an index of the influence of each unit on the outcome of the estimated velocity. We then plotted the weights between congruent/opposite MSTd units and self-/scene-motion units as a function of preferred cue directions, the derivation of which is described in *Multisensory Tuning Properties*. To summarize this data, we binned the weights according to the direction at which they maximally respond.

To further explore the role of congruent/opposite MSTd units, we performed an artificial lesion experiment in which we decoded velocity from either only congruent or opposite units. That is, the activity of particular MSTd units were set to zero while we tested the network’s response to stimuli. Estimates were decoded from all combinations of visual and vestibular inputs ranging from ±4 velocity in 31 linear steps, resulting in 961 estimates. To show the difference between estimates, we normalized the velocity estimates decoded from congruent and opposite units and computed their difference.

## Supplementary Material

Supplementary File

## Data Availability

Data in Figs. 2 and 3 were extracted from published papers (6, 8, 9, 18). We performed analyses in Python using standard packages for numeric and scientific computing. All the code and data used for model optimization and implementations of the optimization procedure is freely and openly available at http://github.com/ReubenRideaux/How-multisensory-neurons-solve-causal-inference and https://osf.io/7n8uj/, respectively.
