# Supplementary material for: How multisensory neurons solve causal inference
Source: Proc Natl Acad Sci U S A. 2021 Aug 4;118(32):e2106235118. doi: 10.1073/pnas.2106235118 (PMC8364184; doi:10.1073/pnas.2106235118)
Supplement: Supplementary File [file pnas.2106235118.sapp.pdf]

Proceedings of the National Academy of Sciences

Supplemental Information

How multisensory neurons solve causal inference

Reuben Rideaux, Katherine R. Storrs, Guido Maiello, and Andrew E. Welchman

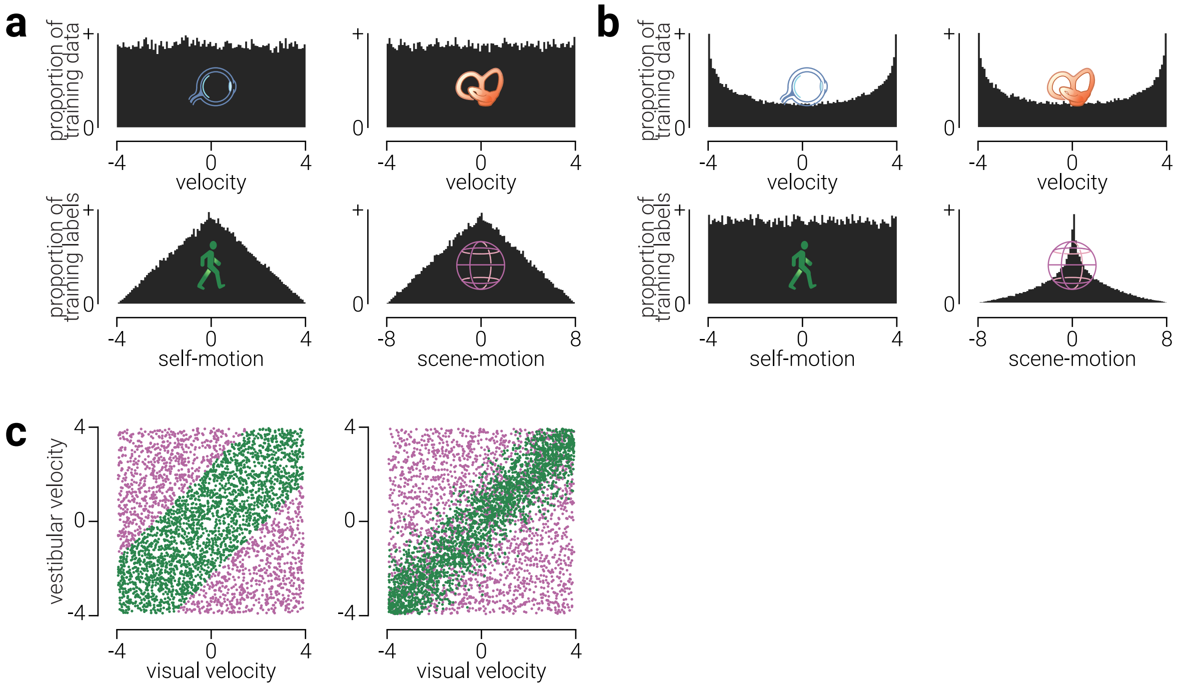

Supplementary Figure 1. Multisensory network training data. **a**) The distributions of (top-left) visual and (top-right) vestibular inputs used to train MultiNet were uniform, and as a result, (bottom-left) self- and (bottom-right) scene-motion solutions were not. It is not possible to have uniform distributions of both self- and scene-motion solutions from the same input pairs (unless all inputs = zero). To test the impact of using a non-uniform distribution of solutions during training, we trained control networks where either the self- or scene-motion solutions were uniform by altering the distribution of inputs. To do so, we calculated the distributions in reverse order. That is, we first generated a uniform distribution of self-/scene-motion solutions, then calculated the input distributions required to produce it. (**b**) shows the distributions for uniform self-motion solutions. **c**, left) Visual and vestibular cues were independently sampled from uniform distributions, and a median split was used to class pairs as either caused by the same (green) or different (magenta) events. Ecologically, there is typically a correlation between signals caused by the same event and no correlation between those caused by different events. To test the impact of using more ecologically valid signal pairs, we simulated same and different events, producing the pattern of pairs shown in (**c**, right). We found no qualitative differences between these control networks and those trained with non-uniform, median-split distributions of solutions for any of the analyses conducted.

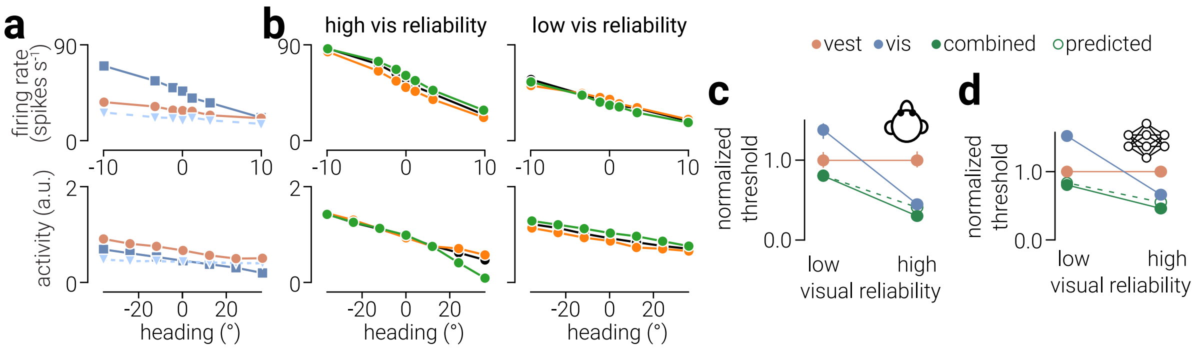

Supplementary Figure 2. Biological correlates of reliability-based cue weighting and cue sensitivity. **a**, top) Mean firing rate of an example macaque MSTd neuron during the single-cue behavioural task shown in the top panel of **Figure 2b**. The top of (**b**) is the same as the top of (**a**), but in response to the combined-cue task shown in (**Figure 2c**, top). The bottom panels in (**a-b**) are the same as the top, but for an example MultiNet MSTd unit. **c**) Predicted (optimal) and observed psychophysical thresholds, normalized to the vestibular threshold; data shown for monkey Y (Fetsch, Pouget, DeAngelis & Angelaki, 2011). **d**) Same as (**c**), but for MultiNet. Data in (a-b [top] and **c**) extracted and replotted from Fetsch et al. (2011).

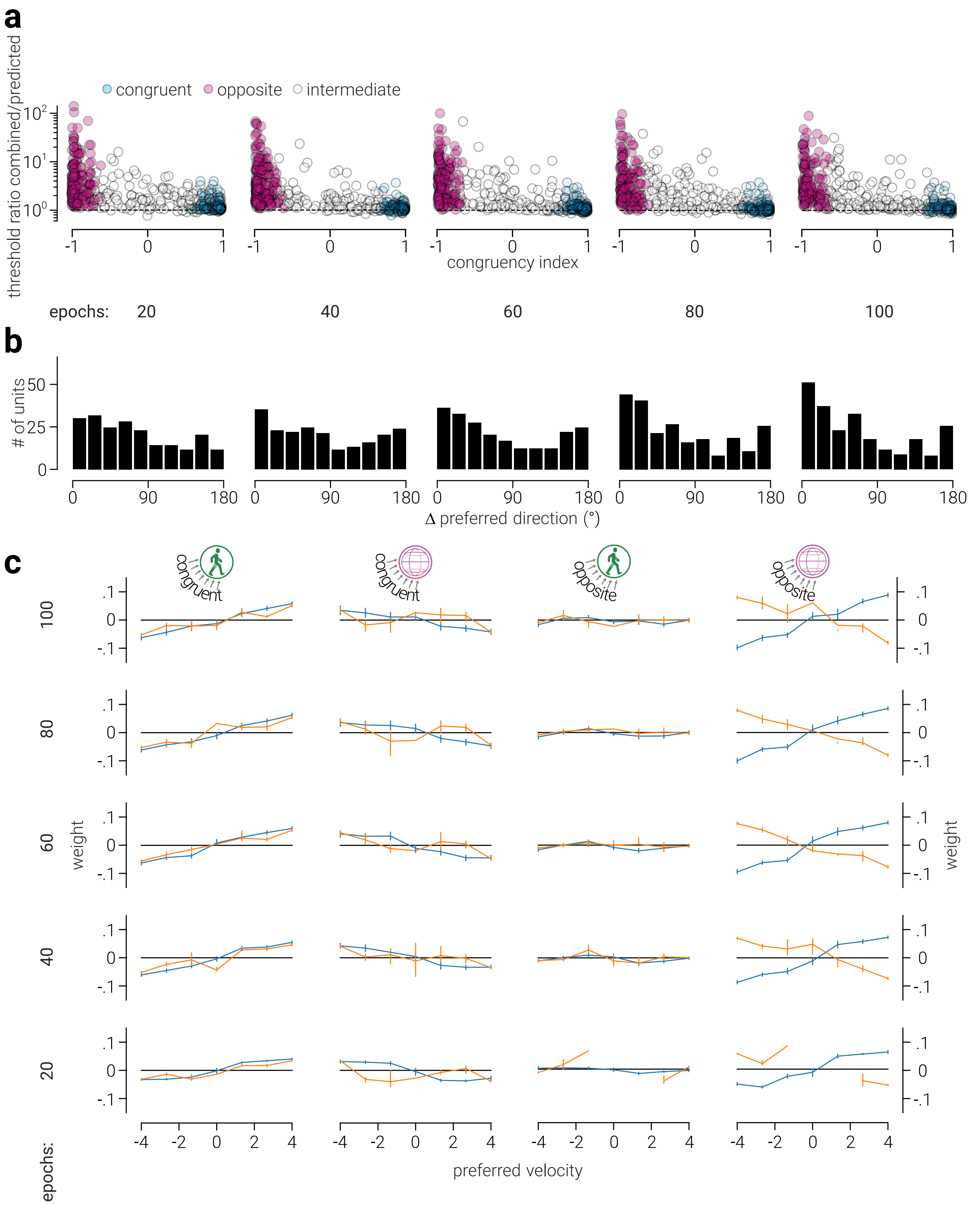

Supplementary Figure 3. Development of multisensory tuning properties across training epochs. **a**) The ratio between the "neuronal" threshold of MSTd units for congruent visual and vestibular cues to that predicted by optimal cue integration as a function of the congruency between their tuning for these cues in isolation. Filled symbols denote neurons for which the congruency index significantly differs from zero. **b**) The distribution of MSTd units as a function of the difference between preferred visual and vestibular heading direction. From left to right, results are shown for networks after 20, 40, 60, 80, and 100 epochs. **c**) The weights shown in **Figure 4c** at different training epochs.

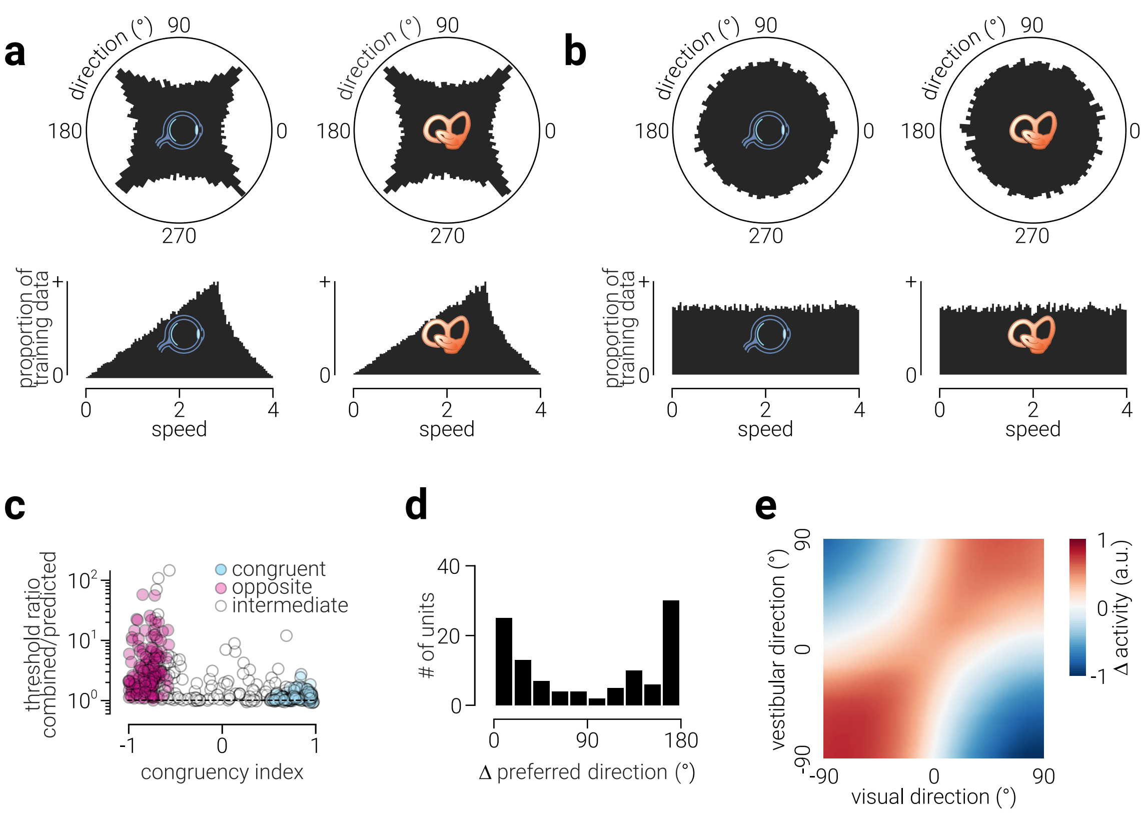

Supplementary Figure 4. Mapping translational, axial, and rotational velocity into polar coordinates. Multinet was trained on inputs drawn from a uniform distribution in cartesian space, (**a**) shows an example of the distribution of motion (top) directions and (bottom) speeds of the training inputs in polar space , e.g., by combining *x* and *y* velocities. **b**) To test the influence of the training on a non-uniform distribution of directions and speeds, we retrained MultiNet, using combinations of v_x and v_z, on a uniform distribution of heading directions. We found the same properties emerged within the network, when trained on a uniform distribution of directions and speeds, showing that the results are not dependent on using a uniform distribution of velocities. Examples results from the network trained on uniform directions and speeds, corresponding to those of MultiNet in **Figures 3d**, **3g**, and **4a**, are shown in (c-e), respectively.

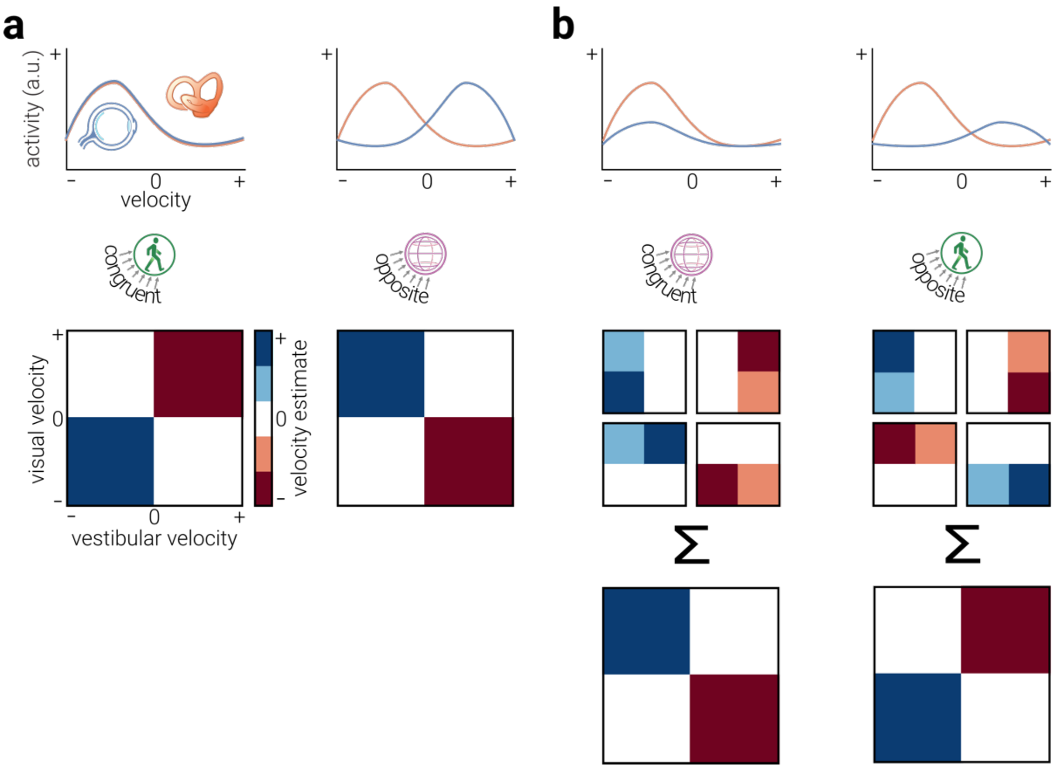

Supplementary Figure 5. Multiple roles of congruent and opposite neurons. **a**) Illustration showing the self- and scene-motion estimates produced by congruent and opposite neurons, respectively, with similar sensitivity to visual and vestibular cues. The upper panels show the tuning to visual and vestibular cues while the lower panels show a coarse representation of the self-/scene-motion velocity estimates by these neurons in response to different combinations of cues; the lower plots use the same mapping as those in **Figure 4d**. **b**) When there is an asymmetry in sensitivity to multisensory cues, congruent and opposite units can, as a subpopulation, reverse their roles by combining counterbalanced outputs that sum to produce scene- and self-motion. That is, individual units show one of four types of connections to outputs units (the smaller plots in the middle), but when their activity is combined (summed) across the population, the result is the pattern of velocity estimates shown in the (large) bottom panels. Thus, individually, all congruent and opposite units behave as expected, promoting self- and scene-motion estimates, respectively. However, as a population, by varying the sensitivity to visual and vestibular cues, congruent and opposite units can flexibly contribute to both estimates.

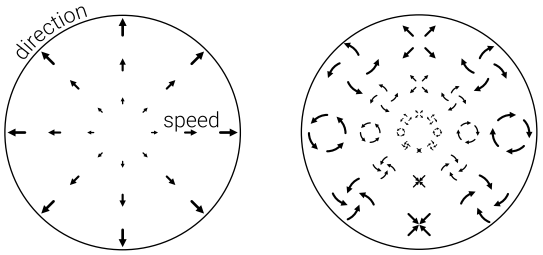

Supplementary Figure 6. Mapping translational, axial, and rotational velocity into polar coordinates. Illustration showing the mapping between (left) translational, i.e., *x* and *y*, velocity in polar coordinates and (right) axial and rotational, i.e., *z* and *r*, velocity into polar coordinates.
